# Effectiveness of Stromal Vascular Fraction (SVF) and Platelet-Rich Plasma (PRP) in Patients With Knee Osteoarthritis: Protocol for a Phase 3, Prospective, Randomized, Controlled, Multicenter Study (SPOST Study)

**DOI:** 10.2196/62659

**Published:** 2025-04-08

**Authors:** Adrien Schwitzguebel, David Andres Ramirez Cadavid, Tamara Da Silva, Pierre Decavel, Charles Benaim

**Affiliations:** 1 Sports Medicine Division Hôpital de la Providence Neuchâtel Switzerland; 2 GALSER SA Neuchâtel Switzerland; 3 Faculté des sciences et de médecine University of Fribourg Fribourg Switzerland; 4 Clinique Romande de Réadaptation Sion Switzerland; 5 Physical Medicine and Rehabilitation division Orthopedic Hospital Centre Hospitalier Universitaire Vaudois Laussanne Switzerland

**Keywords:** stromal vascular fraction, platelet-rich plasma, osteoarthritis, adjuvant therapy, tissue regeneration, clinical efficacy

## Abstract

**Background:**

Available evidence on the conservative treatment of knee osteoarthritis still leaves questions about the efficacy of platelet-rich plasma (PRP) and whether stromal vascular fraction (SVF) offers a superior therapeutic tool.

**Objective:**

This study aims to assess the clinical efficacy of SVF as adjuvant therapy to PRP on functionality and tissue regeneration for knee osteoarthritis.

**Methods:**

In a multicenter, randomized, triple-blind, controlled trial, 108 individuals with knee osteoarthritis will be block-randomized in a 1:1 ratio. Patients will receive an initial single PRP or PRP + SVF injection followed by PRP doses at 1 month and 2 months. The primary endpoint is functional improvement measured with the Western Ontario and McMaster Universities Osteoarthritis Index (WOMAC) at the 6-month follow-up. Secondary endpoints, collected at the 1-month, 2-month, 3-month, 6-month, and 12-month follow-ups, will include the pain visual analogue scale during maximal physical activity, WOMAC score, length of time to return to work and sports in days, magnetic resonance imaging (MRI)–based Whole-Organ Magnetic Resonance Imaging Score (WORMS), Magnetic Resonance Observation of Cartilage Repair Tissue (MOCART) score, MRI Area Measurement and Depth and Underlying Structures (AMADEUS) score at 6 months and at 12 months, adverse events, and serious adverse events.

**Results:**

Participant recruitment and data collection are expected to begin in July 2025 and finish in July 2027. Final end points will be gathered in August 2027, and the results are expected to be published in late 2027.

**Conclusions:**

The study results will provide insight into the clinical efficacy of SVF as adjuvant therapy to PRP on functionality and tissue regeneration in patients with knee osteoarthritis.

**Trial Registration:**

ClinicalTrials.gov (NCT05660824); https://clinicaltrials.gov/study/NCT05660824

**International Registered Report Identifier (IRRID):**

PRR1-10.2196/62659

## Introduction

Osteoarthritis, the most common joint disease [1], has a high social and individual impact, and the development of therapeutic options is a public health priority. Its multifactorial etiology is still a source of active research [2,3]. The most common conservative treatments for osteoarthritis include painkillers, active physical therapies, orthotics, corticosteroid infiltrations, hyaluronic acid, and platelet-rich plasma (PRP) [1,4].

PRP may be beneficial in osteoarthritis by interfering with catabolic and inflammatory events and by subsequently promoting anabolic responses. Activation of PRP releases biologically active components, including platelet-derived growth factor, transforming growth factor-β, type I insulin-like growth factor, and vascular endothelial growth factor. These proteins are responsible for a range of critical tissue healing roles, such as chondrocyte and mesenchymal stem cell (MSC) proliferation, bone and vessel remodeling, inflammatory modulation, and collagen synthesis [5].

Several clinical trials have found improvement in clinical outcomes for osteoarthritis [6,7], presumably associated with the chondroprotective effect of PRP. Nevertheless, despite the numerous studies on the subject, the evidence is inconsistent, there is a lack of uniform improvement in functional outcomes, and an in vivo effect on human cartilage regeneration has not yet been demonstrated [8,9].

Stem cell therapy has arisen as a new therapeutic option for knee osteoarthritis. Preclinical models have elucidated how injected adipose-derived mesenchymal stem cells (AD-MSCs) coordinate the cartilage regeneration process [10-12] through paracrine mechanisms [13], producing cytokines and trophic bioactive factors that stimulate cellular proliferation and reduce inflammation, fibrosis, oxidative stress, and chondrocyte senescence [1].

AD-MSCs seem to have a better hypoxic tolerance, fewer immunologic and inflammatory responses [14], better chondrogenic induction and gene expression [15], and less variable and more reliable clinical result [14] than bone marrow–derived mesenchymal stem cells (B-MSC).

Stromal vascular fraction (SVF), a product from processed adipose tissue, contains MSCs, endothelial precursor cells, T regulatory cells, macrophages, smooth muscle cells, pericytes, and preadipocytes. SVF extraction and injection techniques have recently been used as an alternative to harvest AD-MSCs due to their logistical simplicity and feasibility in clinical practice. The superiority or inferiority of SVF compared with AD-MSC has not yet been established.

Randomized trials indicate that intra-articular SVF injections can provide clinical benefits in knee osteoarthritis [16], with some studies noting cartilage quality improvements [16-19]. Despite their potential, SVF treatments are invasive, costly, and supported by a limited number of studies, many of which lack the homogeneity needed for clinical guideline endorsement, even though research in this area is steadily increasing and yielding promising results. Although PRP is often recommended in sports medicine for knee osteoarthritis, it is unclear whether combining it with SVF offers greater benefits for patients unresponsive to conservative treatment. This randomized controlled trial (RCT) will be the first to provide comparative data on the efficacy of SVF as an adjunct to PRP, addressing a critical gap in osteoarthritis treatment research.

The objectives of this study are to assess the clinical efficacy of SVF as adjuvant therapy to PRP on (1) functionality for knee osteoarthritis and (2) tissue regeneration for knee osteoarthritis.

## Methods

### Study Design

This multicenter, parallel-group, triple-blind study will enroll 108 patients who will be randomly assigned in a 1:1 ratio to either the intervention (SVF+PRP injection at baseline) or control group (PRP-only injection at baseline) using stratified randomization. The study will use a superiority framework.

The follow-up will last 12 months, with endpoints at 1 month, 2 months, 3 months, 6 months, and 12 months.

### Study Setting

In this multicenter study, we aim to recruit all patients between July 2025 and August 2027 in the Sports Medicine Division of La Providence Hospital and in the Rehabilitation division, Hôpital Fribourgeois, both in Switzerland. The lead center is the Sports Medicine Division of La Providence Hospital where author AS is the sponsor-investigator. All the interventions will be performed in the lead center.

### Eligibility Criteria

Patients will be recruited if they have knee osteoarthritis with persistent symptoms despite appropriate first-line treatment (ie, active physical therapies, sport and daily activity adaptations, orthotics use, medication) and for whom a surgical procedure is not indicated nor recommended.

The following main inclusion criteria will be used: (1) age older than 16 years; (2) symptomatic knee osteoarthritis confirmed by magnetic resonance imaging (MRI); (3) absence of free or displaced meniscal or cartilage fragments on the MRI of the affected knee; and (4) failure of first-line conservative management, including medical or infiltrative treatment, orthotics use, active rehabilitation plan, and adaptation of sports and work habits, in the last 3 months.

The following main exclusion criteria will be used: (1) patients familiar with the lipoaspiration process; (2) significant disease of the contralateral member with a disability, as evaluated with a Western Ontario and McMaster Universities Osteoarthritis Index (WOMAC) score >80%; (3) co-existence of microcrystalline disease (ie, gout, pseudogout); (4) active inflammatory rheumatic disorders; (5) a need for regular anti-inflammatory treatment (either nonsteroidal anti-inflammatory drugs [NSAIDs] or corticosteroids) or anticoagulants; (6) patients with decompensated renal failure, hepatic dysfunction, or severe pulmonary or cardiovascular disease; (7) patients with an immunocompromised status; and (8) women who are pregnant or intend to become pregnant during the study.

If bilateral disease is present and both sides require either the experimental or control intervention, only the most symptomatic side will be studied.

Informed consent will be obtained by the local site investigator. Other physicians in associated structures (ie, orthopedists, general practitioners, physiotherapists) are informed of the study and will be asked to refer patients to the referent sports medicine departments.

### Interventions

In both study groups, the intervention will be performed in the operating room under aseptic conditions and following the Arthrex protocols to prepare the autologous conditioned plasma [20], which is our PRP, and autologous conditioned adipose [21], which is our SVF, as described in the following paragraphs.

The patient is placed with a surgical drape hiding the interventional zone from the patient’s sight. The blinded investigator performs a 1.5 mm incision under local anesthesia on each abdomen side in order to introduce the microcannula used to extract the SVF. Tumescent solution is prepared by mixing 500 mL NACL with 30 cc 2% lidocaine + adrenaline 1:200000 and 3 cc 8.4% sodium bicarbonate, then 60 mL of this preparation are injected into each side and left in place. An interval of 20 minutes is allowed, which is necessary to let the tumescent solution act on the abdominal adipose tissue. At this point, the blinded investigator leaves the room. In the experimental arm, the unblinded investigator uses a double syringe system to extract 30 mL adipose tissue from the abdominal incisions. This is then centrifugated at 2500 rpm for 4 minutes. The oil and water are discarded. The lipoaspirate is then filtered using two 20 mL syringes and a 1.4-mm diameter transfer hub. One last centrifugation is performed at 2500 rpm for 4 minutes obtaining 2 mL to 6 mL SVF and the remaining oil. This last mixture is then discarded. In the control arm, the unblinded investigator performs a sham adipose tissue extraction by introducing the extraction cannula and moving it for 2 minutes on each side. In both study arms, the venipuncture is then performed, and 15 mL blood are extracted for the PRP preparation using the Arthrex double syringe system [20]. The blood is then centrifuged at 1500 rpm for 5 minutes, resulting in 4 mL to 7 mL of PRP. No anticoagulant, calcium chloride, nor other PRP activator is added. Following the aseptic technique, the unblinded investigator prepares the SVF-PRP mixture or PRP alone in an opaque 10-mL syringe. In parallel, the patient is prepared for the ultrasound-guided injection procedure. With an 18-gauge needle, the SVF-PRP mixture or PRP alone is then injected by the blinded investigator.

Postintervention care includes (1) partial weight-bearing for 1 month; (2) active strengthening of the muscles without overloading the knee using, if possible, the blood-flow restriction technique and gentle nonweight-bearing muscle activation, including core stability exercises; (3) mobility; and (4) modification of daily activities, work, and sports habits.

**Figure 1:**
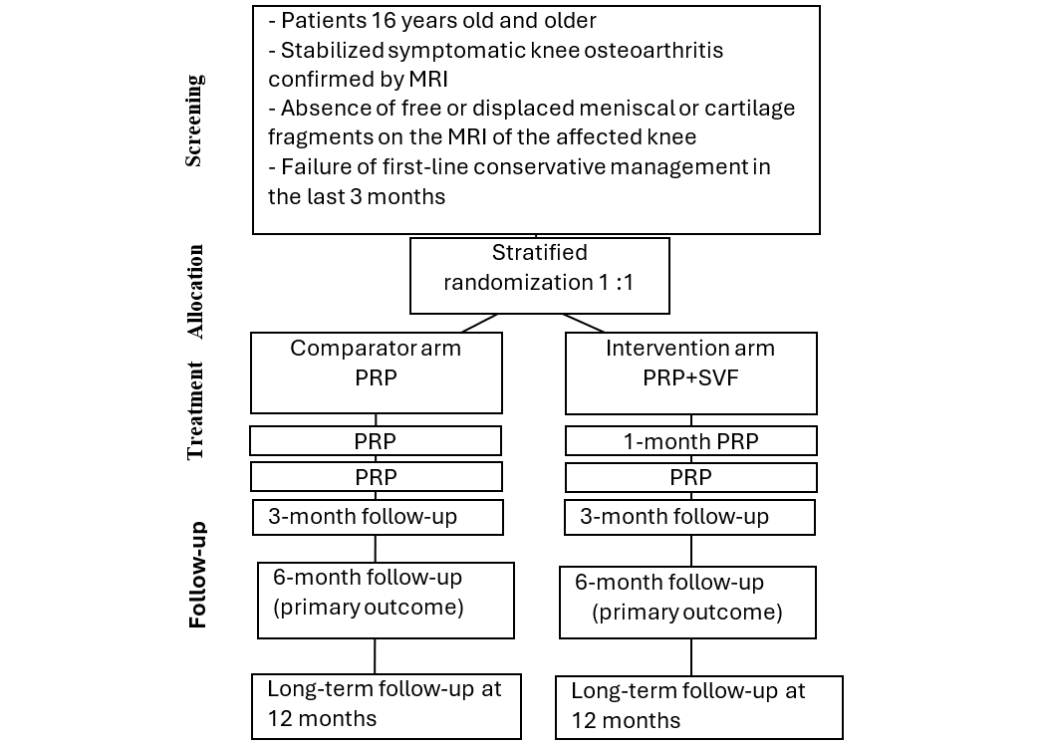
Study flow chart. MRI: magnetic resonance imaging. PRP: platelet-rich plasma.

A total of 5 follow-up visits are planned for the study, during which principal and secondary outcomes will be gathered by a blinded investigator. At the 1-month and 2-month follow-up visits, patients in both study arms will receive 2 additional ultrasound-guided PRP injections performed by the blinded investigator and following the same Arthrex autologous conditioned plasma technique. The same postintervention care will be provided after these 2 PRP injections. Figure 1 shows the study flow chart.

Anticoagulants (eg, aspirin) and anti-inflammatory drugs (eg, ibuprofen, naproxen, meloxicam) should not be used 2 weeks before and 2 weeks after each injection as it can potentially interrupt the therapeutic acute inflammatory response and cytokine production.

Active physical therapies regimens, orthotics use, sports, and daily activities are adapted to the patient, on a day-by-day basis, during the postintervention care.

### Outcome Measurements and Assessments

The primary outcome is functional improvement measured with the 0%-100% normalized WOMAC at the 6-month follow-up, where 0% indicates complete absence of symptoms and 100% indicates maximal possible symptom severity. A clinically relevant functional improvement is a difference of 9.1 points out of 100 points.

The secondary outcomes will be clinical and radiological parameters gathered at the 1-month, 2-month, 3-month, 6-month, and 12-month follow-ups. These include a 10-point pain visual analogue scale (VAS) during maximal physical activity performed by the patient according to manageable pain and clinical recommendations, the 0%-100% normalized WOMAC [2], length of time to return to work and sports in days, adverse events (AEs), and serious adverse events (SAEs). Pain will be assessed as an AE of interest, during the intervention and 48 hours following the intervention.

The improvement in cartilage quality will be assessed at the 6-month and 12-month follow-ups (previous MRI should not be dated more than 3 months before the intervention) using 3 key MRI-based scoring systems: Area Measurement and Depth and Underlying Structures (AMADEUS) [13], Whole-Organ Magnetic Resonance Imaging Score (WORMS) [22], and Magnetic Resonance Observation of Cartilage Repair Tissue (MOCART) [23].

We initially planned to statistically assess these outcomes at 6 months and 12 months. However, assessment of these parameters at other time points might be subject to exploratory analysis.

The participant timeline is presented in Table 1. The following information will be collected at baseline: age, gender, height, weight, BMI, smoking status, comorbidities, baseline Kellgren–Lawrence grade, current and previous treatments, and posttraumatic etiology. The set of clinical and radiological scores include the VAS, WOMAC, return to work and sports in days, AMADEUS, WORMS, and MOCART.

**Table 1:** Study assessments and procedures at the study visits.

Procedures	Screening^a^	Baseline	Visit 1	Visit 2	Visit 3	Visit 4	Visit 5
Timing (visit window)	–3 weeks (+3 weeks)	0	1 month (±7 days)	2 months (±7 days)	3 months (±7 days)	6 months (±7 days)	12 months (±7 days)
Informed consent	X	—^b^	—	—	—	—	—
Inclusion/exclusion criteria	X	—	—	—	—	—	—
Baseline characteristics	—	X	—	—	—	—	—
Clinical scores	—	X	X	X	X	X	X
MRI^c^	—	X	—	—	—	X	X
Randomization	—	X	—	—	—	—	—
Lipoharvesting or sham lipoharvesting	—	X	—	—	—	—	—
Intervention^d^	—	X	—	—	—	—	—
PRP^e^ injection	—	—	X	X	—	—	—
Concomitant medication	—	X	X	X	X	X	X
AEs^f^ and SAEs^g^	—	X	X	X	X	X	X

^a^Screening and baseline visits can be performed on the same day if the patient has been given reasonable time to make a consented decision about participation in the study.

^b^—: not applicable.

^c^MRI: magnetic resonance imaging.

^d^Stromal vascular fraction+platelet-rich plasma (PRP) or PRP-only injection depending on the group.

^e^PRP: platelet-rich plasma.

^f^AEs: adverse events.

^g^SAEs: serious adverse events.

### Sample Size

The sample size for this study was calculated using PS software (Vanderbilt University) [24]. We aimed to detect a clinically meaningful difference of 9.1% in the absolute change in the WOMAC function score between the experimental and control groups. The calculation assumed an SD of 13.9%, based on findings from the study conducted by Tubach et al [25].

In their study, Tubach et al [25] evaluated changes in the WOMAC function score in patients with knee osteoarthritis treated with NSAIDs over a 4-week period. They reported an average baseline WOMAC function score of 42.8 (SD 16.1) and an absolute improvement of –11.6 (SD 13.9). These values were used to estimate the variability in absolute changes for our calculation.

**Figure 2:**
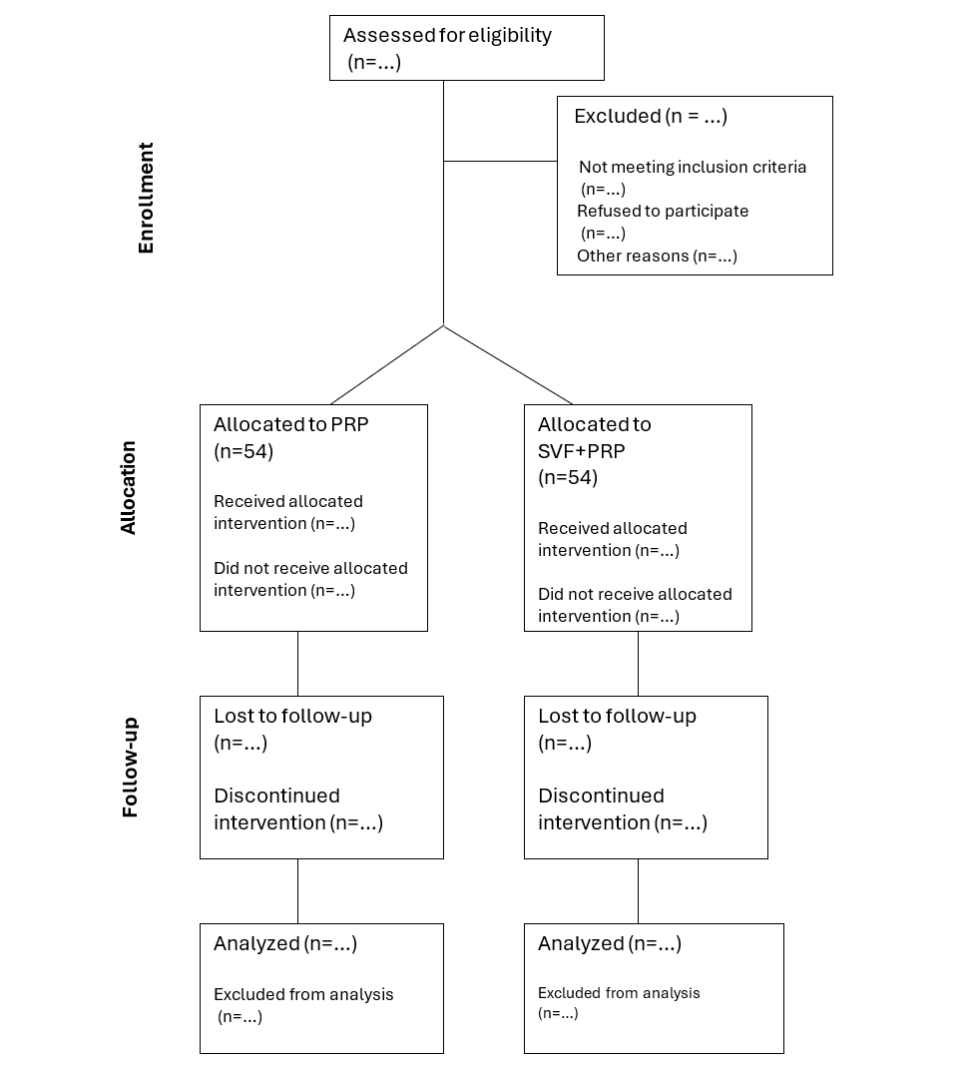
CONSORT (Consolidated Standards of Reporting Trials) diagram showing the flow of participants through each stage of the randomized trial. PRP: platelet-rich plasma; SVF: stromal vascular fraction.

With these parameters, a sample size of 108 patients (54 per group) was determined to provide 90% power to detect the specified difference at a 5% significance level using a 2-tailed test. This calculation ensures that our study is adequately powered to detect a meaningful treatment effect based on absolute improvements in functional outcomes. The study will collaborate with health care providers in both study centers to achieve the target sample size. Additionally, potential participants will be contacted through patient registries and referrals. All recruitment efforts will comply with ethical guidelines and prioritize informed consent. Figure 2 shows the CONSORT (Consolidated Standards of Reporting Trials) diagram [26].

### Randomization and Blinding

Study participants will be randomized at a 1:1 ratio to either the intervention arm or the active comparator arm using a randomization table and blocked randomization with blocks of 4 patients. Participants will be assigned to specific strata based on age (older or younger than 40 years) and the presence of cartilage defects (partial, full, or full with bone deformation).

The unblinded investigator responsible for the study interventions will perform the randomization shortly before administering the interventions. This study is triple-blinded: (1) Participants will be unaware of their group assignment throughout the intervention and study duration, (2) the investigator performing the intraarticular injection and the outcome assessor will remain blinded, and (3) the statistician will also be blinded. The investigator responsible for preparing the PRP+SVF or PRP-only preparation will be unblinded and will not participate in outcome assessments.

All patients will undergo a venipuncture, either a lipoaspirate or a sham lipoaspirate, followed by an ultrasonographically guided PRP or PRP+SVF injection. It is anticipated that unblinding will not be required to ensure patient safety, as no known adverse effects of the SVF (such as local pain, tenderness, hematoma on donor site, or infection) require unblinding for clinical management. However, if unblinding becomes necessary due to unexpected circumstances, the unblinded investigator will disclose the allocated intervention for the affected patients.

### Data Collection and Management

All data collected in this study will be recorded in standardized electronic case report forms (CRFs; see Multimedia Appendices 1-4). We plan to use Hermes software. Hermes is a tool developed by sponsor-investigator AS, used for a patient registry, and validated by the local ethics committee board (authorization # CERVD AO_2020-00006). Only data with logical numeric variables within the correct ranges or predefined categorical variables can be entered into the electronic CRFs. Data entry for variables of interest is mandatory.

CRFs will be kept current to reflect participant status at each phase during the course of the study. Study-related data will be collected in a coded manner (participants will not be identified in the CRF by name or initials). Identification of patients must be guaranteed at the study site. Patients’ identifications will be recorded in a sequential list stored in the local investigator’s secured server. At the end of the study, when the database has been checked for completeness and validated by the sponsor-investigator, it will be locked and used for statistical analyses. All “study essential documents” (eg, informed consent form, CRFs) will be archived for at least 10 years after completion of the clinical trial.

Data sharing is not applicable to this article, as, to date, no data sets have been generated nor analyzed.

### Statistical Analysis

The WOMAC score will be normalized to a 0%-100% scale, where 0% indicates no symptoms and 100% represents the highest severity of symptoms. This normalization involves converting the raw scores into percentages, facilitating the interpretation and comparison of results across participants and study groups. The absolute difference at 6 months will serve as the primary outcome and will be compared using either the paired Student *t* test or the Wilcoxon signed-rank test, depending on the distribution of the variable, with intention-to-treat analysis. The absolute differences in secondary outcomes (changes from baseline to other time points) between the treatment and control groups will be evaluated with the appropriate statistical test (categorical variables: chi-square or Fisher exact tests; continuous variables: Student *t* or Wilcoxon rank tests). All analyses will include both intention-to-treat and per-protocol analyses. Effect estimates, 95% CIs, and descriptive *P* values will be reported whenever possible, along with corresponding graphs.

A post hoc analysis will attempt to identify variables of interest using the appropriate global linear model.

Since the intervention is considered low risk, no interim analysis is planned. In case of missing primary outcome data, patients will be withdrawn from the analysis. Patients missing secondary outcome data will remain in the study but be excluded from those specific analyses.

### Oversight and Monitoring

For quality control of the study conduct and data retrieval, all study sites will have regular monitoring activities performed by appropriately trained and qualified monitors, who are outsourced by the sponsor-investigator. Monitoring activities will consist of on-site monitoring as well as remote and centralized monitoring.

The objectives of a monitoring visit are to (1) verify the informed consent form process for each monitored participant, (2) verify the prompt and accurate recording of all monitored data points and prompt reporting of all safety events, (3) compare collected data with participants’ source documents, and (4) ensure investigators comply with the protocol.

The monitors may also inspect the clinical site regulatory files to ensure that regulatory requirements and applicable guidelines (International Council for Harmonisation [ICH] Good Clinical Practice [10]) are being followed.

SAEs will be defined as any untoward medical occurrence that results in death, is life-threatening, requires inpatient hospitalization or prolongation of existing hospitalization, results in persistent or significant disability or incapacity, or in a congenital anomaly or birth defect. SAEs should be followed until resolution or stabilization. Assessment of causality will be based on the criteria listed in the ICH E2A guidelines [10], and severity will be graded based on the Common Terminology Criteria for Adverse Events Version 5 [27]. All SAEs will be reported immediately and within a maximum of 24 hours to the sponsor-investigator of the study. The sponsor-investigator will re-evaluate the SAE and return the form to the co-investigator. SAEs resulting in death will be reported via the sponsor-investigator to the Swiss Business Administration System for Ethics Committees and to the other ethics committees involved in the trial within 7 days.

Regular audits are not planned. For the purpose of on-site inspection or audit, the competent authorities or ethics committee may require access to all source documents and other study-related records. The sponsor-investigator and local investigators must ensure the availability of these documents at any time.

### Ethical Considerations

#### Ethics Review Approvals

To comply with local regulations in Switzerland regarding clinical trials involving human subjects, this study design is classified as risk category C [28-30].

Therefore, both Swissmedic (the Swiss authority responsible for the authorization and supervision of therapeutic products) and the local ethics committee must review and approve the research protocol. Since both applications require a significant investment, a grant request will be submitted prior to applying to these two institutions. The principal investigator will obtain approval from the competent authority (Swissmedic) before the start of the study. Once the protocol is approved, no changes will be made without prior approval from the ethics committee, except when necessary to eliminate immediate hazards to participants.

#### Informed Consent

The recruiting investigator will explain the study's nature, purpose, procedures, duration, risks, benefits, and discomforts. Participation will be voluntary, and participants can withdraw at any time without affecting their medical care. Each participant will receive an information sheet and consent form to make an informed decision, with time to consult others and ask questions. Consent will be obtained before any procedures, and the signed form will be kept as part of the records. Participants will be informed that authorized individuals may examine their medical records. The informed consent is available in Multimedia Appendix 5.

#### Privacy and Confidentiality

The investigator upholds the participant's right to privacy and complies with privacy laws, ensuring anonymity in scientific presentations and publications. Medical information from the study is confidential, and third-party disclosure is prohibited. Participant confidentiality will be maintained using identification code numbers. Authorized representatives, such as those from Swissmedic or the ethics committee, may access relevant medical records for data verification.

#### Compensation Details

There is no compensation provided for participants in this human subjects research.

#### Identifiable Features

Identifiable features of research participants in any image or supplementary material will not be visible.

## Results

This version of the study protocol presented in this article is ready to be presented to the Switzerland regulatory authorities. A request for funding was submitted to the Swiss Medical Foundation in December 2024, and the study was registered in the portal for clinical trials in Switzerland (number pending).

Enrollment to the study is expected to begin in July 2025 and finish in July 2027. Final end points will be gathered in August 2027, and the results are expected to be published in late 2027. The findings will be shared through conference presentations targeting rehabilitation and sports medicine specialists. The Swiss Medical Network and Switzerland's Physical Medicine and Rehabilitation Network will be engaged through presentations at their yearly research events to help implement findings in clinical settings.

Given the open-access nature of the target journal, all results will be publicly available. To gather feedback, we will use postpublication surveys, interactive webinars, and follow-up interviews with patients and practitioners to discuss the applicability and impact of SVF therapy in practice.

## Discussion

This study design intends to assess the potential benefits of SVF injection, an easy-to-use, simple, and noninvasive cellular therapy, on the most frequent joint disease. With this high-quality RCT, SVF will be compared with one of the most commonly used noninvasive therapies in the field of sports medicine, PRP.

Based on available clinical trials, we hypothesize that SVF treatment in addition to PRP may lead to significant clinical and radiological improvement in patients with knee osteoarthritis. The WOMAC and VAS scores are among the most commonly used clinical outcomes in RCTs and meta-analyses for knee osteoarthritis research. For radiological assessment, 3 MRI-based scoring systems—AMADEUS, MOCART, and WORMS—are frequently cited in RCTs. Including these scores as primary and secondary outcomes will facilitate future meta-analyses and allow direct comparison of our trial’s results with those of previous studies

Compared with PRP, cellular therapy with SVF requires more physician training, takes more time, and ultimately incurs higher costs. In other words, it demands greater resources. From the authors’ perspective, patients and caregivers should only invest in these additional resources if clear benefits are demonstrated, which this study aims to establish.

Different techniques have been used to inject or implant cellular therapy with MSCs to the required site, but consensus about the best approach does not exist. However, biologically, some elements support the use of AD-MSCs for nonbone tissue. AD-MSCs would theoretically be more resilient than B-MSCs to the hypoxic articular cavity because they are less dependent on mitochondrial respiration for energy production. From an immunological perspective, AD-MSCs should be preferable to B-MSCs since B-MSCs could induce a higher immunological response due to their higher cell-surface human leukocyte antigen class I expression. AD-MSC highly expresses interleukin-33, which promotes regulatory T cell phenotype proliferation, which would theoretically mean a beneficial effect on anti-inflammatory responses [14].

Han et al [11] compared AD-MSC with B-MSC for knee osteoarthritis and found a superior therapeutic effect of AD-MSC compared with B-MSC on VAS and WOMAC scores. Zhou et al [31] performed a meta-analysis comparing AD-MSC and B-MSC therapies, and they found no statistical differences in clinical scores between the two therapies but did find a higher variability in B-MSC results, suggesting AD-MSCs might be a more reliable therapeutic option. Ude et al [32] found better chondrogenic inductions and gene expressions with AD-MSCs than with B-MSCs.

The therapeutic potential of MSCs is of great interest due to their possible long-term chondroprotective and even chondroregenerative effects. A meta-analysis by Lee et al [19] demonstrated significant improvements in imaging outcomes measured using the WORMS and MOCART scores. WORMS scores improved significantly with SVF therapy compared with controls at both 6 months and 12 months posttreatment (6 months: mean difference [MD]=–18.29, 95% CI – 21.75 to –14.84; 12 months: MD=–26.78, 95% CI –29.95 to –23.61). Similarly, MOCART scores showed notable improvements with AD-MSC therapy at 6 months (MD=24.7, 95% CI 5.92 to 43.48) and 24 months (MD=25.8, 95% CI 5.52 to 46.08), while SVF therapy resulted in significant gains at 6 months, 12 months, and 24 months (6 months: MD=30.11, 95% CI 26.08 to 34.13; 12 months: MD=36.82, 95% CI 23.95 to 49.68; 24 months: MD=10.60, 95% CI 1.37 to 19.83).

The comparative superiority of SVF over AD-MSCs, or vice versa, remains uncertain, as no RCT has yet directly addressed this question. An observational study [33] suggested the superiority of AD-MSCs, but further evidence is needed.

The meta-analysis by Lee et al [19] showed some differences between both therapies on clinical outcomes. A significant improvement in the VAS score was found at 6 months and 12 months with AD-MSCs (6 months: MD=–1.62, 95% CI –2.46 to –0.79; 12 months: MD=–1.97, 95% CI –3.22 to –0.72), whereas SVF showed significant results only at 12 months (6 months: MD=–2.32, 95% CI –5.15 to 0.52; 12 months: MD=–2.13, 95% CI –3.06 to –1.21). In contrast, functionality measured with WOMAC scores improved significantly 6 months after SVF treatment (MD=–6.12, 95% CI –10.71 to 1.52), while AD-MSC therapy yielded significant improvements only after 12 months (6 months: MD=–1.96, 95% CI –5.36 to 1.45). At 12 months and 24 months, both SVF and AD-MSC therapies produced significant improvements in the WOMAC score (12 months SVF: MD=–9.09, 95% CI –12.67 to –5.51; 12 months AD-MSC: MD=–9.19, 95% CI –12.48 to –5.90; 24 months SVF: MD=–10.71, 95% CI –18.49 to –2.93; 24 months AD-MSC: MD=–6.88, 95% CI –10.24 to –3.52).

The first main strength of our study design is the large sample size. Second, our study population is well-designed and reproducible, with standardized diagnostic criteria and failure of a first-line standardized rehabilitation plan. Third, the multicenter design allows for better reproducibility of patient selection and management, even if interventions are performed at the main study center by a single investigator. This is, however, a study strength, as it will avoid bias related to the intervention techniques.

A sham lipoaspiration procedure with the patient awake is performed to maintain blinding in the control group. Despite all precautions taken to uphold allocation concealment, breaches may occur if the patient diligently questions the procedure. For ethical reasons, if at 6 months, the progression is unsatisfactory, the intervention's nature may be disclosed if deemed clinically relevant, allowing the patient to benefit from SVF infiltration. Consequently, the study may be limited, as the initial 12-month secondary outcomes will not be considered in the final analysis. Finally, the patient selection, clinical follow-up, and rehabilitation plan might differ across the various recruitment centers (multicenter design).

The minimal clinically important difference was chosen as the limit to detect a clinically relevant difference for sample size calculation. From the authors’ point of view, even in the case of statistically significant positive effects of the treatment, the generalization of the procedure should be balanced by a complementary cost-effectiveness analysis. Indeed, in case of mild benefits, other procedures such as strengthening, biomechanics adaptation, medication, annual PRP infiltrations, shockwave therapies, or even slight daily activity adaptations might be options of choice. One should be aware that cellular therapies should not be presented to patients as a “magic potion” or “youth elixir.” Especially, the authors warn about the recognized risk of financial benefits based on overemphasized clinical promises.

This study will contribute data to establish the clinical relevance of SVF treatment for the most relevant disease of the musculoskeletal system.

## Appendix

Multimedia Appendix 1 Participant Screening Case Report Form (CRF).

Multimedia Appendix 2 Participants Enrollment Case Report Form (CRF).

Multimedia Appendix 3 Participants Follow-up Case Report Form (CRF).

Multimedia Appendix 4 Averse Events and Serious Adverse Events Case Report Form (CRF).

Multimedia Appendix 5 Patients informed consent.

## Abbreviations

AD-MSC: adipose-derived mesenchymal stem cells
AE: adverse event
AMADEUS: Area Measurement and Depth and Underlying Structures
B-MSC: bone marrow–derived mesenchymal stem cells
CONSORT: Consolidated Standards of Reporting Trials
CRF: case report form
ICH: International Council for Harmonisation
MD: mean difference
MOCART: Magnetic Resonance Observation of Cartilage Repair Tissue
MRI: magnetic resonance imaging
MSC: mesenchymal stem cell
NSAID: nonsteroidal anti-inflammatory drug
PRP: platelet-rich plasma
RCT: randomized controlled trial
SAE: serious adverse events
SVF: stromal vascular fraction
VAS: visual analog scale
WOMAC: Western Ontario and McMaster Universities Osteoarthritis Index
WORMS: Whole-Organ Magnetic Resonance Imaging Score

## Data Availability

All data requests should be directed to the corresponding author.
