# Supplementary material for: Effectiveness of Stromal Vascular Fraction (SVF) and Platelet-Rich Plasma (PRP) in Patients With Knee Osteoarthritis: Protocol for a Phase 3, Prospective, Randomized, Controlled, Multicenter Study (SPOST Study)
Source: JMIR Res Protoc. 2025 Apr 8;14:e62659. doi: 10.2196/62659 (PMC12015334; doi:10.2196/62659)
Supplement: Multimedia Appendix 1 [file resprot_v14i1e62659_app1.pdf]

Effectiveness of Stromal Vascular Fraction (SVF) and Platelets Rich Plasma (PRP) in patients with knee Osteoarthritis: Study protocol for a phase III, prospective, randomized, controlled multi-center study: (SPOST study).

Case Report Form (CRF)

Screening CRF

LA PROVIDENCE
Médecine du sport

AS médecine du sport

SCREENING CRF

Inclusion criteria

Informed consent as documented by signature:

- [ ] Filled
- [ ] Not filled

Symptomatic osteoarthritis of the hip, knee, ankle, elbow or shoulder confirmed by MRI.

- [ ] Filled
- [ ] Not filled

Over 16 years old.

- [ ] Filled
- [ ] Not filled

Unchanged treatment protocol for ALL of the following: Medications, Infiltrations, Use of orthotics, Rehabilitation plan, Complementary therapies, Sports and work habits

- [ ] Filled
- [ ] Not filled

Failure of previous infiltrative treatment
(corticosteroids, platelet-rich plasma, visco-supplementation)

- [ ] Filled
- [ ] Not filled

Proven absence of pregnancy with adequate contraception or negative pregnancy test & absence of desire to procreate

- [ ] Filled
- [ ] Not filled

No current anti-inflammatory treatment (either NSAIDs or corticoids)

Filled

Not filled

Absence of decompensated renal failure, liver dysfunction or severe pulmonary or cardiovascular disease

Filled

Not filled

No active inflammatory rheumatic disorders

Filled

Not filled

No immunosupresion

Filled

Not filled

No allergy to local anesthetics or epinephrine

Filled

Not filled

Absence of coagulation disorders or anticoagulant treatment

Filled

Not filled

No inability to follow study procedures, e.g. due to language problems, psychological disorders, dementia, etc. of the participant,

Filled

Not filled

No drug or alcohol abuse

Filled

Not filled

No registration prior to current study

Filled

Not filled

No participation in any other study with an investigational drug or procedure in the 30 days preceding and during the present study.

Filled

Not filled

The patient is not a member of the group of investigators, family members, employees or other dependents.

Filled

Not filled

Western Ontario and McMaster Universities Osteoarthritis Index (WOMAC) score

How bad is the pain?
When walking on a flat surface?

No

Minime

Moderate

Severe

Very severe

How bad is the pain?
When you go up or down the stairs?

- No
- Minime
- Moderate
- Severe
- Very severe

How bad is the pain?
At night, when you're in bed?

- No
- Minime
- Moderate
- Severe
- Very severe

How bad is the pain?
When you stand up from a chair or sit down?

No

Minime

Moderate

Severe

Very severe

How bad is the pain?
When you stand?

No

Minime

Moderate

Severe

Very severe

How difficult is it for you to :
Going down the stairs?

No

Minime

Moderate

Severe

Very severe

LA PROVIDENCE
Médecine du sport

AS médecine du sport

How difficult is it for you to :
Climbing stairs?

- No
- Minime
- Moderate
- Severe
- Very severe

How difficult is it for you to :
Do you want to get up from a sitting position?

- No
- Minime
- Moderate
- Severe
- Very severe

How difficult is it for you to :
Stand on your own two feet?

- No
- Minime
- Moderate
- Severe
- Très severe

How difficult is it for you to :

Leaning forward?

- No
- Minime
- Moderate
- Severe
- Très severe

How difficult is it for you to :

Walking on flat ground?

- No
- Minime
- Moderate
- Severe
- Très severe

How difficult is it for you to :

Getting in and out of a car?

- No
- Minime
- Moderate
- Severe
- Très severe

LA PROVIDENCE
Médecine du sport

AS médecine du sport

How difficult is it for you to :
Shopping?

No

Minime

Moderate

Severe

Très severe

How difficult is it for you to :
Tights or socks?

No

Minime

Moderate

Severe

Très severe

How difficult is it for you to :
Out of bed?

No

Minime

Moderate

Severe

Très severe

How difficult is it for you to :
Take off your tights or socks?

- No
- Minime
- Moderate
- Severe
- Très severe

How difficult is it for you to :
Lie down on the bed?

- No
- Minime
- Moderate
- Severe
- Très severe

How difficult is it for you to :
Getting in or out of a bathtub?

- No
- Minime
- Moderate
- Severe
- Très severe

How difficult is it for you to :
Would you like to sit down?

No

Minime

Moderate

Severe

Très severe

How difficult is it for you to :
Getting in and out of the toilet?

No

Minime

Moderate

Severe

Très severe

How difficult is it for you to :
Deep clean your home?

No

Minime

Moderate

Severe

Très severe

How difficult is it for you to :
Daily maintenance of your home?

No

Minime

Moderate

Severe

Très severe

How stiff is your joint?
When you get up in the morning?

No

Minime

Moderate

Severe

Très severe

How stiff is your joint?
When you move around after sitting, lying down or resting during the day?

No

Minime

Moderate

Severe

Very severe
