# Supplementary material for: Effectiveness of Stromal Vascular Fraction (SVF) and Platelet-Rich Plasma (PRP) in Patients With Knee Osteoarthritis: Protocol for a Phase 3, Prospective, Randomized, Controlled, Multicenter Study (SPOST Study)
Source: JMIR Res Protoc. 2025 Apr 8;14:e62659. doi: 10.2196/62659 (PMC12015334; doi:10.2196/62659)
Supplement: Multimedia Appendix 2 [file resprot_v14i1e62659_app2.pdf]

Effectiveness of Stromal Vascular Fraction (SVF) and Platelets Rich Plasma (PRP) in patients with knee Osteoarthritis: Study protocol for a phase III, prospective, randomized, controlled multi-center study: (SPOST study).

**Case Report Form (CRF)**

**CRF Enrollment**

# ENROLLMENT CRF

## Baseline characteristics

**Age**

[ ] Years

**Gender**

- Male
- female

**Height**

[ ] cms

**Weight**

[ ] Kg

**Tobacco consumption**

- No
- Yes

**Presence of comorbidities?**

- Diabetes mellitus
- Osteoporosis
- Dyslipidemia
- presence of rheumatological disease
- Hypertension
- kidney failure
- Osteopenia
- other relevant comorbidity?

**Date of onset of symptoms**

dd.mm.yyyy

**Post-traumatic etiology?**

- Yes
- No
- Perhaps

**Current and previous treatment of osteoarthritis**

- non-steroidal anti-inflammatory drugs: NSAID
- Paracetamol
- Opioid analgesics
- Steroid injections
- Hyaluronic acid Injections
- Selective serotonin reuptake inhibitors (SSRIs) Which ones? *
- Platelet-rich plasma PRP
- Shock waves
- US Therapeutics
- Which surgery? *
- Physiotherapy
- Eccentric exercises
- Other treatment / specify treatment (medical, dosage, etc.)

# Clinical results

**VAS score: last month**

_______ /10

**SANE**

_______ %

**Stopped sporting activities?**

- yes
- no

**Stopped work?**

- yes
- no

# PRE-INTERVENTION RADIOLOGY REPORT

**Kellgren-Lawrence grade.**

- 0: Normal X-ray
- 1: Possible narrowing of joint space with or without osteophytes
- 2: Clear osteophytes, no or slight joint narrowing
- 3: Moderate osteophytes and joint narrowing possible sclerosis deformity
- 4: Large osteophytes Marked narrowing of joint space Severe sclerosis Deformity

# AMADEUS Score

Area measurement on MRI

**Defect size**

- No defect
- ≤ 1cm2
- >1 ≤2 cm2
- >2 ≤4 cm2
- >4 ≤6 cm2
- >6 cm2

**Defect depth**

- (n) No defect
- (a) signal alteration
- (b) Partial tickness defect
- (c) full thickness defect

Underlying structures on MRI

**Subchondral bone defect**

A. No defect

B. bony defect/ cyst ≤ 5 mm depth

C. bony defect/ cyst > 5 mm depth

**Addendum-potential fourth digit**

No defect-associated bone-marrow oedema

Defect-associated bone-marrow oedema

# MOCART Score (Magnetic Resonance Observation of Cartilage Repair Tissue)

### 1. Degree of defect repair and filling of the defect

Complete (100%)

Hypertrophy (greater than 100%)

Incomplete (50-100%)

Incomplete (less than 50%)

Subchondral bone exposed

### 2. Integration to border zone

Complete

Incomplete

No integration

### 3. Surface of the repair tissue

- Intact
- Damage less than 50% of the repair tissue depth
- Damage more than 50% of the repair tissue depth
- Subchondral bone exposed

### 4. Structure of the repair tissue

- Homogeneous
- Inhomogeneous

### 5. Signal intensity of the repair tissue

- Isointense
- Moderately hyperintense
- Severely hyperintense

### 6. Subchondral lamina

- Intact
- Not intact

### 7. Subchondral bone

- Intact
- Edema
- Cyst formation

LA PROVIDENCE
Médecine du sport

AS médecine du sport

### 8. Adhesions

Present

Absent

### 9. Effusion

Present

Absent

# WORMS Score (Whole Organ Magnetic Resonance Imaging Score)

### 1. Cartilage Morphology (0-6 per compartment)

Normal thickness and signal

1: Normal thickness but increased signal on T2-weighted images

2: Partial-thickness focal defect <1 cm in greatest width

3: Multiple areas of partial-thickness defects or a grade 2 lesion wider than 1 cm

4: Full-thickness focal defect <1 cm in greatest width

5: Multiple areas of full-thickness defects or a grade 4 lesion wider than 1 cm, but less than 75% of the region

6: Diffuse (≥ 75% of the region) full-thickness loss

### 2. Bone Marrow Abnormalities (0-3 per compartment)

Normal

1: Mild (small lesion <1 cm)

2: Moderate (lesion 1-2 cm)

3: Severe (lesion >2 cm)

### 3. Subchondral Cysts (0-3 per compartment)

| |
|---|
| 0: No cysts |
| 1: Small cysts (≤5 mm) |
| 2: Medium cysts (>5 mm and ≤10 mm) |
| 3: Large cysts (>10 mm) |

### 4. Bone Attrition (0-3 per compartment)

| |
|---|
| 0: None |
| 1: Mild flattening or surface irregularity |
| 2: Moderate flattening |
| 3: Severe flattening or deformity |

### 5. Osteophytes (0-7 per site)

| |
|---|
| 0: None |
| 1: Mild (≤ 2 mm) |
| 2: Moderate (2-5 mm) |
| 3: Large (>5 mm) |

LA PROVIDENCE
Médecine du sport

AS médecine du sport

**6. Effusion (0-3)**

0: None

1: Mild

2: Moderate

3: Severe

**7. Meniscal Abnormalities (0-4 per meniscus)**

0: None

1: Intrasubstance abnormalities

2: Non-displaced tear

3: Displaced tear or partial maceration

4. Complete maceration or destruction
