# Supplementary material for: Effectiveness of Stromal Vascular Fraction (SVF) and Platelet-Rich Plasma (PRP) in Patients With Knee Osteoarthritis: Protocol for a Phase 3, Prospective, Randomized, Controlled, Multicenter Study (SPOST Study)
Source: JMIR Res Protoc. 2025 Apr 8;14:e62659. doi: 10.2196/62659 (PMC12015334; doi:10.2196/62659)
Supplement: Multimedia Appendix 3 [file resprot_v14i1e62659_app3.pdf]

Effectiveness of Stromal Vascular Fraction (SVF) and Platelets Rich Plasma (PRP) in patients with knee Osteoarthritis: Study protocol for a phase III, prospective, randomized, controlled multi-center study: (SPOST study).

Case Report Form (CRF)

Follow-up CRF

LA PROVIDENCE
Médecine du sport

AS médecine du sport

Clinical Follow-up

Visit number: Time:

Visit N 4: 1 month post-intervention: Follow-up N 1

Visit N 5: 2 months post-intervention: Follow-up N 2

Visit N 6: 3 months post-intervention: Follow-up N 3

Visit N 7: 6 months post-intervention: Follow-up N 4

Visit N 8: 12 months post-intervention: Follow-up N 5

Clinical outcomes

VAS score: last month

/10

SANE: last month

%

yes

no

Has the patient resumed sporting activities? YES/ NO

Date of resumption of sporting activities?

dd.mm.yyyy

Has the patient resumed work activities? YES/NO

Date of resumption of work activities

dd.mm.yyyy

Global Rating Score

As for your (joints, arms, elbows, ankles, knees, etc.), how are they generally doing?

-3

-2

-1

0

+1

+2

+3

Western Ontario and McMaster Universities Osteoarthritis Index (WOMAC) score

How bad is the pain?
When walking on a flat surface?

- No
- Minime
- Moderate
- Severe
- Very severe

How bad is the pain?
When you go up or down the stairs?

- No
- Minime
- Moderate
- Severe
- Very severe

How bad is the pain?
At night, when you're in bed?

- No
- Minime
- Moderate
- Severe
- Very severe

How bad is the pain?
When you stand up from a chair or sit down?

- No
- Minime
- Moderate
- Severe
- Very severe

How bad is the pain?
When you stand up?

- No
- Minime
- Moderate
- Severe
- Very severe

How difficult is it for you to :
Going down the stairs?

- No
- Minime
- Moderate
- Severe
- Very severe

How difficult is it for you to :
Climbing stairs?

- No
- Minime
- Moderate
- Severe
- Very severe

How difficult is it for you to :
Do you want to get up from a sitting position?

- No
- Minime
- Moderate
- Severe
- Very severe

How difficult is it for you to :
Stand on your own two feet?

- No
- Minime
- Moderate
- Severe
- Very severe

How difficult is it for you to :

Leaning forward?

- No
- Minime
- Moderate
- Severe
- Very severe

How difficult is it for you to :

Walking on flat ground?

- No
- Minime
- Moderate
- Severe
- Very severe

How difficult is it for you to :

Getting in and out of a car?

- No
- Minime
- Moderate
- Severe
- Very severe

How difficult is it for you to :
Shopping?

- No
- Minime
- Moderate
- Severe
- Very severe

How difficult is it for you to :
Tights or socks?

- No
- Minime
- Moderate
- Severe
- Very severe

How difficult is it for you to :
Out of bed?

- No
- Minime
- Moderate
- Severe
- Very severe

How difficult is it for you to :
Take off your tights or socks?

- No
- Minime
- Moderate
- Severe
- Very severe

How difficult is it for you to :
Lie down on the bed?

- No
- Minime
- Moderate
- Severe
- Very severe

How difficult is it for you to :
Getting in or out of a bathtub?

- No
- Minime
- Moderate
- Severe
- Very severe

How difficult is it for you to :
Would you like to sit down?

No

Minime

Moderate

Severe

Very severe

How difficult is it for you to :
Getting in and out of the toilet?

No

Minime

Moderate

Severe

Very severe

How difficult is it for you to :
Deep clean your home?

No

Minime

Moderate

Severe

Very severe

How difficult is it for you to :
Daily maintenance of your home?

- No
- Minime
- Moderate
- Severe
- Very severe

How stiff is your joint?
When you get up in the morning?

- No
- Minime
- Moderate
- Severe
- Very severe

How stiff is your joint?
When you move around after sitting, lying down or resting during the day?

- No
- Minime
- Moderate
- Severe

Rehabilitation results :

Number of physiotherapy sessions

Self-reported compliance. Number of self-exercise sessions per week

1	5
2	6
3	7
4	>7

Need for subsequent treatments

- Shock wave therapy
- Corticosteroids
- Platelet-rich plasma
- Visco-supplementation
- Complementary medicine
- No

Number of days of orthotic use per week

Use of orthoses after injection.

< 1	5
2	6
3	7
4	

Deviations to the protocol

Deviation to the rehabilitation protocol

Yes

No

New concomitant therapy

NSAIDs, corticotherapy, surgery, ...

Yes

No

Other informations

Eventual comments

comorbidity, new problem, ...

Post-procedure radiological follow-up

AMADEUS Score

Area measurement

- No defect
- ≤ 1cm2
- >1 ≤2 cm2

Defect size

- >2 ≤4 cm2
- >4 ≤6 cm2
- >6 cm2

Defect depth

- (n) No defect
- (a) signal alteration
- (b) Partial tickness defect
- (c) full thickness defect

Underlying structures on MRI

Subchondral bone defect

- A. No defect
- B. bony defect/ cyst ≤ 5 mm depth
- C. bony defect/ cyst > 5 mm depth

Addendum-potential fourth digit

- No defect-associated bone-marrow oedema
- Defect-associated bone-marrow oedema

MOCART Score (Magnetic Resonance Observation of Cartilage Repair Tissue)

1. Degree of defect repair and filling of the defect

- Complete (100%)
- Hypertrophy (greater than 100%)
- Incomplete (50-100%)
- Incomplete (less than 50%)
- Subchondral bone exposed

2. Integration to border zone

- Complete
- Incomplete
- No integration

3. Surface of the repair tissue

- Intact
- Damage less than 50% of the repair tissue depth
- Damage more than 50% of the repair tissue depth
- Subchondral bone exposed

4. Structure of the repair tissue

- Homogeneous
- Inhomogeneous

5. Signal intensity of the repair tissue

- Isointense
- Moderately hyperintense
- Severely hyperintense

6. Subchondral lamina

- Intact
- Not intact

7. Subchondral bone

- Intact
- Edema
- Cyst formation

LA PROVIDENCE
Médecine du sport

AS médecine du sport

8. Adhesions

- Present
- Absent

9. Effusion

- Present
- Absent

WORMS Score (Whole Organ Magnetic Resonance Imaging Score)

1. Cartilage Morphology (0-6 per compartment)

- Normal thickness and signal
- 1: Normal thickness but increased signal on T2-weighted images
- 2: Partial-thickness focal defect <1 cm in greatest width
- 3: Multiple areas of partial-thickness defects or a grade 2 lesion wider than 1 cm
- 4: Full-thickness focal defect <1 cm in greatest width
- 5: Multiple areas of full-thickness defects or a grade 4 lesion wider than 1 cm, but less than 75% of the region
- 6: Diffuse (≥ 75% of the region) full-thickness loss

2. Bone Marrow Abnormalities (0-3 per compartment)

- Normal
- 1: Mild (small lesion <1 cm)
- 2: Moderate (lesion 1-2 cm)
- 3: Severe (lesion >2 cm)

LA PROVIDENCE
Médecine du sport

AS médecine du sport

3. Subchondral Cysts (0-3 per compartment)

0: No cysts
1: Small cysts (≤5 mm)
2: Medium cysts (>5 mm and ≤10 mm)
3: Large cysts (>10 mm)

4. Bone Attrition (0-3 per compartment)

0: None
1: Mild flattening or surface irregularity
2: Moderate flattening
3: Severe flattening or deformity

5. Osteophytes (0-7 per site)

0: None
1: Mild (≤ 2 mm)
2: Moderate (2-5 mm)
3: Large (>5 mm)

LA PROVIDENCE
Médecine du sport

AS médecine du sport

6. Effusion (0-3)

0: None

1: Mild

2: Moderate

3: Severe

7. Meniscal Abnormalities (0-4 per meniscus)

0: None

1: Intrasubstance abnormalities

2: Non-displaced tear

3: Displaced tear or partial maceration

4. Complete maceration or destruction
