# Supplementary material for: Effectiveness of Stromal Vascular Fraction (SVF) and Platelet-Rich Plasma (PRP) in Patients With Knee Osteoarthritis: Protocol for a Phase 3, Prospective, Randomized, Controlled, Multicenter Study (SPOST Study)
Source: JMIR Res Protoc. 2025 Apr 8;14:e62659. doi: 10.2196/62659 (PMC12015334; doi:10.2196/62659)
Supplement: Multimedia Appendix 4 [file resprot_v14i1e62659_app4.pdf]

Effectiveness of Stromal Vascular Fraction (SVF) and Platelets Rich Plasma (PRP) in patients with knee Osteoarthritis: Study protocol for a phase III, prospective, randomized, controlled multi-center study: (SPOST study).

Case Report Form (CRF)

Adverse Events CRF
Serious Adverse Event CRF

ADVERSE EVENT CRF

Registre d'évènement indésirable

Semaines depuis l'intervention

[ ] Semaines

Degré de sévérité

- Légère, grade 1
- Moderé, grade 2
- Grave, grade 3
- Grade 4
- Grade 5

Nature

Decrivez la nature de l'evenement indesirable

Traitement entrepris:

- Aucune
- Medicament(s)
- Autre

Peut l'evenement indesirable être en relation avec l'intervention d'étude?

- Exclu
- pas exclu (effet indésirable médicamenteux-ADR-)
- non évaluable

Si ADR, médicament d'étude stoppé ?

- Oui
- Non

Issue :

- En cours
- Résolu
- Résolu avec séquelles
- Inconnue

Nom de l'investigateur et site qui fait le repport d'evenement indésirable

LA PROVIDENCE
Médecine du sport

AS médecine du sport

SERIOUS ADVERSE EVENT CRF

Semaines depuis l'intervention

[ ] Semaines

Degré de sévérité

- Légère, grade 1
- Moderé, grade 2
- Grave, grade 3
- Grade 4
- 5. Grade 5

Evénement indésirable grave (SAE)

Nature

Decrivez la nature de l'evenement indesirable

Cocher la case correspondante :

- Décès (quelle que soit la cause)
- Etat critique, pouvant entraîner la mort
- Hospitalisation ou prolongation de l'hospitalisation
- Séquelles durables (ex : cancer, malformations,...)
- Invalidité, handicap transitoire ou non
- Intérêt médical particulier (ex: convulsions, développement d'une dépendance à la drogue ou abus de drogue,...)

L'évenement indésirable grave est-il attendu ?

- Oui
- Non

Degré de causalité du médicament d'étude (imputabilité):

Certain (1)

Probable (2)

Possible (3)

Improbable (unlikely) (4)

Non évaluable (unclassifiable) (5)

Traitement entrepris:

aucun

médicament(s)

☐ Autre

lequel?

Si ADR (effet indésirable médicamenteux), médicament d'étude stoppé ?

Oui

Non

Issue:

Rétablissement

Aggravation

Amélioration

Séquelles

Stabilisation

Mort

En cours

Inconnue

Nom de l’investigateur qui fait le repport d'evenement indésirable
