# Supplementary material for: Effectiveness of Stromal Vascular Fraction (SVF) and Platelet-Rich Plasma (PRP) in Patients With Knee Osteoarthritis: Protocol for a Phase 3, Prospective, Randomized, Controlled, Multicenter Study (SPOST Study)
Source: JMIR Res Protoc. 2025 Apr 8;14:e62659. doi: 10.2196/62659 (PMC12015334; doi:10.2196/62659)
Supplement: Multimedia Appendix 5 [file resprot_v14i1e62659_app5.pdf]

Application for participation in a medical research project :

Can the injection of stem cells from belly fat improve osteoarthritis of the knee that doesn't respond to other treatments?

Dear Sir or Madam

We invite you to take part in our research project.

Your participation is entirely voluntary. All data collected as part of this project is subject to strict data protection regulations.

The research project is being carried out by Dr. Adrien Schwitzguébel and his team at the Hôpital de la Providence and the team at the Hôpital de Fribourgeois. We'll let you know the results if you wish.

We'll be happy to discuss the essentials with you and answer any questions you may have. To give you an overview of the project, here are the key points to bear in mind. More detailed information follows.

Why are we conducting this research project?

In the presence of knee osteoarthritis, the first line of treatment is rehabilitation, with the aim of relieving pain and hoping for recovery if the lesions are not too advanced. Rehabilitation treatment includes physiotherapeutic therapy, with adaptation of sporting and occupational activities, the use of support splints, and pain-relieving medication or infiltration. If rehabilitation fails, surgery can sometimes be proposed, but at the cost of potential side-effects and complications.

Our research project aims to study whether the efficacy of injecting stem cells from your belly fat in addition to platelet-rich plasma is effective in the event of insufficient results from rehabilitative treatment.

What should I do if I agree to participate? - What happens to me if I participate?

Form of participation: If you agree to take part in our project, we will remove a small amount of abdominal fat, extract the stem cells and reinject them with PRP (Platelet Rich Plasma) into the knee joint. One group of patients will receive the injection of stem cells and PRP, and another group will receive PRP only.

Procedure for participants: On the same day, under local anaesthetic, we will take fat from your belly and process it to select stem cells. At the same time, we'll withdraw 15ml of venous blood, which we'll process to prepare the PRP. Then we'll inject either the stem cells with PRP, or PRP alone into your knee joint.

30 and 60 days after the first treatment, we will repeat the preparation of the PRP, also from your blood, and inject it into the knee joint.

Duration: The first treatment lasts about an hour, with the next two PRP injections lasting about 30 minutes.

Number of consultations, time required and associated constraints: Post-infiltration medical follow-up lasts 12 months, with medical consultations scheduled before infiltration, then at 1, 2, 3, 6 and 12 months post-infiltration.

What are the benefits and risks of participating in the project?

Benefits for participants

Your participation in this project can directly benefit you by partially or totally curing your osteoarthritis or tendonitis problem.

By taking part, you are helping to support future patients.

Risks and constraints

The procedure is unpleasant and painful, despite a well-managed local anaesthetic.

With the available scientific evidence, we know that the risks associated with the procedure include pain at the site of fat extraction and injection of stem cells and PRP, local inflammatory reaction with possible fluid in the joint, in rare cases infection (potentially serious), haematoma or bleeding, serious unanticipated side effects (extremely unlikely).

By signing at the end of the document, you certify that you have fully understood its contents and freely consent to take part in the project.

Detailed information

1. Project objective and selection of participants

In this information sheet, our research project is referred to simply as a "study". If you agree to take part, you will be considered a study participant.

Before a therapeutic procedure can be used to treat a disease, it must be scientifically evaluated in a study involving participants. The aim of this project is to examine and measure the efficacy, tolerance and safety of the injection of stem cells derived from abdominal fat (commercial kit used: ACA Arthrex® for "autologous conditioned adipose tissue") as well as the tolerance and safety of the injection of Platelet Rich Plasma (commercial kit used: ACP Arthrex® for "autologous conditioned plasma") in the treatment of knee osteoarthritis resistant to standard rehabilitation treatments.

The stem cell preparation, called ACA, has the following characteristics: 1.5 to 5 ml of this preparation contain "stem cells", or more precisely "pericytes", i.e. progenitor cells capable of transforming into other cell types. Injected in the right place, these cells can become either cartilage cells or supporting cells producing substances necessary for the healing of damaged cartilage. This procedure has already been performed many times, with over 2,000 uses listed in the scientific literature.

The second preparation, called ACP Arthrex, is a process commonly used in sports medicine and other fields, such as aesthetic medicine. This process, authorized and frequently used in Switzerland, is derived from your own blood and contains between 1 and 5 ml of plasma, with very few red blood cells, very few white blood cells, and around one million platelets. The platelets will bring back growth factors that can act on the biology of your cartilage, helping to improve your condition.

We'd like to hear from you, as participation is open to all those suffering from knee osteoarthritis who are not responding to well-managed rehabilitation treatment

2. General information about the project

We are conducting this research project to determine whether there is an advantage to receiving an ACA injection to the knee, in addition to 3 PCA injections, versus receiving only the 3 PCA injections. The aim is to relieve your pain and, to some extent, repair the cartilage.

- ACA injections are authorized in Switzerland only if the fat removed and treated is reinjected into fat. The use we are planning, i.e. the injection of ACA into the knee joint space, is therefore not currently authorized, except in the context of a clinical study such as this one. This procedure is authorized and routinely used in the United States.
- Intra-articular ACP injections are authorized in Switzerland.
- In this study, all participants will receive 3 injections of ACP: at the start of the study, then at 1 and 2 months. All patients will also undergo abdominal fat extraction. One group of participants will receive ACA treatment, while the other will not. Treatment efficacy will be measured by comparing pain, perceived knee functionality and cartilage improvement on MRI.
- After extracting 30 ml of abdominal fat, we prepare the ACA by centrifuging and filtering the extracted volume twice, thus obtaining a variable volume of preparation, generally between 1.5 and 5 millilitres.
- To prepare the PCR, we start with a 15 ml blood sample. The blood will be centrifuged to extract a platelet-rich concentrate, which will be placed in an opaque syringe, with or without ACA.
- If you are assigned to the "ACA" group, you will receive the injection of both products. If you are assigned to the "ACP" group, you will receive only the ACP. Neither Dr. Schwitzguébel nor the doctors in charge of your follow-up will know which treatment you have received.
- Under ultrasound guidance, Dr. Schwitzguébel administers a local anaesthetic, then injects the contents of the opaque syringe.
- After the injection, you will be monitored for 12 months. Our study is "multicentric", i.e. it will take place at two sites: Hôpital de la Providence (Neuchâtel) and Hôpital de Fribourg. All infiltrations will be performed at Hôpital de la Providence. We expect to include 108 patients.

- This study is carried out in compliance with Swiss legislation. We are also following all internationally recognized guidelines. The competent ethics commission and Swissmedic have reviewed and authorized the study.
- A description of the study can also be found on the website of the Swiss Federal Office of Public Health: www.kofam.ch (Study # XXXXXXX).

3. Procedure for participants

Visit	Planning	Treatment	Follow-up 1	Follow-up 2	Follow-up 3	Follow-up 4	Follow-up 5
Timing		0 months	1 month	2 months	3 months	6 months	12 months
Medical consultation	x		x	x	x	x	x
Intervention		x	x	x			
Imaging for study purposes						*x*	*x*
Data collection for study purposes	*x*		*x*	*x*	*x*	*x*	*x*

The table above summarizes the progress of the study. Please find in italics the specific research needs for which we seek your collaboration:

- During each visit, we'll ask you a few simple questions and then calculate a functional score, so that we can best assess the impact of your problem on your functioning.

- At 6 and 12 months post-infiltration, we will perform an MRI of the knee to compare the pre- and post-infiltration condition.

We may have to withdraw you from the study before the scheduled end date. This situation may arise in the event of unforeseen circumstances compromising the scientific validity of the results. In this case, for your own safety, we will offer to examine you one last time. Your continued medical care is guaranteed at all times.

Your doctor will be informed of your participation in this study.

4. Benefits for participants

Participating in the study may bring you a direct benefit by partially curing your osteoarthritis problem and improving your functionality and pain. But you may not benefit at all. The results of the study could prove important in the future for people affected by the same disease

5. Voluntary participation and obligations

Your participation is entirely voluntary. If you choose not to participate, or if you choose to participate and change your mind during the course of the study, you will not have to justify your decision. This will not affect your usual medical care.

If you choose to participate in the study, you will be required to :

- follow the instructions and meet the requirements of the research protocol;
- keep your physician-investigator informed of the progress of your illness, and report any new symptoms, disorders or changes in your condition;
- to inform the physician-investigator of any treatment or therapy, whether traditional or complementary, whether prescribed by another physician or therapist or self-medicated.

6. Risks and constraints for participants

- The procedure is unpleasant, even painful, despite a well-administered local anaesthetic.

- With the available scientific evidence, we know that the risks associated with the procedure include pain at the site of fat extraction and injection of stem cells and PRP, local inflammatory reaction with possible fluid in the joint, in rare cases infection (potentially serious), haematoma or bleeding, undiagnosed serious side effects (extremely unlikely).
- You will have to undergo 2 MRI scans of the knee, which can be unpleasant, especially if you suffer from claustrophobia.

For women of childbearing potential

The data collected to date indicate that the substance tested is unlikely to harm the unborn child. Moreover, there is no evidence to suggest any effect on pregnancy. However, the effects on the human foetus have not yet been sufficiently studied to make clear statements. For this reason, participants should use simple, acceptable contraception for the first 6 months of the study

- Preparation containing progesterone,
- Condoms with or without spermicide,
- Contraceptive cap, diaphragm or sponge with spermicide.

If you discover that you are pregnant during the study, you must inform the investigating physician immediately. In this case, you will be asked to provide information on the progress and outcome of the pregnancy. The investigating physician will discuss the appropriate course of action with you.

7. Alternatives

Participation in the study involves both benefits and risks. If you are asked to take part in the study, this means that you have already been offered or tested other non-surgical treatment options. The physician-investigator will advise you on this during the interview.

8. Results

The study yields various results:

1. Individual results that concern you directly,

2. Individual results discovered by chance (so-called fortuitous discoveries),
3. The final, objective results of the study as a whole.

1. The investigating physician will notify you during the study of any important new findings concerning you. You will be informed orally and in writing; you will then be able to decide again whether you wish to continue your participation in the study.

2. Incidental findings are "concomitant findings", i.e. findings that were not explicitly sought, but were obtained by chance. This might include, for example, the results of a follow-up MRI at 6 and 12 months post-infiltration with ACA, revealing a problem unrelated to the ACA treatment.

You will be informed of chance discoveries if they have an impact on your health. This means that you will be informed of such discoveries if a previously unknown pathology is discovered by chance, or if the onset of a disease can be prevented by preventive measures. If you do not wish to receive this information ("right not to know"), please let the physician-investigator know.

3. At the end of the study, the physician-investigator can send you a summary of the overall results.

9. Data and sample confidentiality

9.1. Data processing and coding

As part of this study, personal and health data are collected and processed, in part automatically. This information is coded at the time of collection. Coding means that all identifying data (name, date of birth, etc.) is replaced by a code (coding accepted by Swissethics). It is not possible to link the data to your person without the code, which remains permanently within Providence Hospital

Only a limited number of people may consult your data in unencrypted form, and only in order to carry out the tasks required for the study. These persons are bound by professional secrecy. As a participant, you have the right to access your data

9.2. Data and sample protection

All data protection guidelines are strictly adhered to. Your data may have to be transmitted in coded form, e.g. for publication, and may be made available to other researchers.

The people in charge of your medical care may be contacted about your state of health.

9.3. Consultation rights during inspections

The study may be subject to inspection. These may be carried out by the relevant ethics commission, or by the Swiss authority for the control and authorization of therapeutic products, Swissmedic. In such cases, the physician-investigator must disclose your data for the purposes of these inspections. All those involved are bound by the strictest professional secrecy.

10. Project withdrawal

You may withdraw from the study at any time. However, medical data and biological material collected up to that point may still be analyzed in coded form.

In the event of withdrawal, your data and samples will continue to appear in coded form in study documents, primarily to ensure medical safety. You must therefore agree to this before giving your consent.

11. Compensation

You will not receive any compensation for your participation in this study

Your participation will have no financial consequences for you or your health insurance. In other words, you will not be billed for information gathering, treatment or imaging tests not required for your clinical follow-up. On the other hand, clinical follow-up will be billed, regardless of whether you participate in or withdraw from the study.

In some cases, the results of this project may help to develop commercial products (e.g. drugs). If you agree to take part in this study, you also waive any right to commercial exploitation (particularly under patents).

12. Liability

Dr. Adrien Schwitzguébel (sponsor), who initiated the research project and is responsible for its implementation, is liable for any damage you may suffer in connection with the study procedure or research activities (e.g. examinations). The conditions and procedure are laid down by law. Dr. Adrien Schwitzguébel (Hôpital de la Providence, Faubourg de l'hôpital 81, 2000 Neuchâtel) has taken out an insurance policy with Zurich Tecta Versicherung (Mythenquai 2 8002 Zurich) to cover any damage for which he is liable.

Should you suffer any damage as a result of your participation in the study, you should contact the physician-investigator mentioned above

13. Financing

The study is funded by XXXXX. (Funding is currently being sought).

14. Contact person(s)

You can ask questions about the study at any time. If you have any doubts, concerns or emergencies during or after the study, you can contact one of the following people:

Dr Adrien Schwitzguébel

Providence Hospital

Faubourg de l'hôpital 81, 2000 Neuchâtel

Secretariat: 032 720 32 75

Direct line, emergencies only: 079 762 05 62

Declaration of consent

Written consent for participation in a clinical study

Please read this form carefully. Don't hesitate to ask questions if you don't understand something or need clarification. Your written consent is required to participate in the project.

**BASEC number of the research project (after submission to the relevant ethics commission) :**	XXXXXXX
**Title:**	Efficacy of stromal vascular fraction (SVF) and platelet-rich plasma (PRP) in patients with osteoarthritis of the knee: study protocol for a multicenter, prospective, randomized, controlled phase III study: (SPOST study)
**Institution responsible (promoter and full address) :**	Dr Adrien Schwitzguébel:<br>Providence Hospital<br>Faubourg de l'hôpital 81, 2000 Neuchâtel
**Location :**	Hôpital de la Providence (Neuchâtel)
**Physician-investigator responsible for the site :**<br>Print name and surname :	
**Participant :**<br>Printed name and surname :<br>Date of birth :	

- I, the undersigned physician-investigator, declare that I have been informed orally and in writing of the objectives and progress of the study involving infiltration of ACA and ACP, as well as the possible advantages, disadvantages and risks.
- I am taking part in this study voluntarily and accept the contents of the information sheet I have been given. I have had sufficient time to make my decision.
- I have received answers to the questions I asked in connection with my participation in this study. I will keep the information sheet and receive a copy of my written declaration of consent.
- I have been informed of the therapeutic alternatives to the project, e.g. the existence of other treatments and therapies.
- I agree that my treating physician may be informed of my participation in the study.
- I agree that the competent specialists of the promoter of this project, of the competent ethics commission and of the Swiss authority for the control and authorization of therapeutic products Swissmedic may consult my uncoded data in order to carry out controls and inspections, on condition however that the confidentiality of these data is strictly ensured.
- I will be informed of the results and/or any incidental findings that have a direct impact on my health. If I do not wish to receive this information, I will contact the physician-investigator.
- If applicable : I am aware that my personal data, health data (and samples) may be transmitted for research purposes within the scope of this study and only in coded form. The sponsor ensures data protection in accordance with Swiss standards and requirements.
- I may revoke my consent to participate in the study at any time and without having to justify myself, without this decision having any adverse repercussions on my further treatment. However, the data and biological material collected up to that point will be analyzed as part of the study.
- I have been informed that Dr Adrien Schwitzguébel has taken out insurance to cover any damage I may suffer as a result of the project.
- I am aware that the obligations mentioned in the participant information sheet must be respected throughout the study. The physician-investigator may exclude me from the study at any time in the interest of my health.

Place, date	Signature of participant

Attestation by physician-investigator: I hereby attest that I have explained the nature, importance and scope of the study to the participant. I declare that I have fulfilled all my obligations in connection with this study in accordance with applicable Swiss law. Should I become aware, at any time during the course of the study, of any factors that might affect the participant's consent to take part in the project, I undertake to inform him/her immediately.

Place, date	Print name and surname of physician-investigator.<br><br><br>Signature of physician-investigator
